# More than words: can free reports adequately measure the richness of perception?

**DOI:** 10.1093/nc/niae035

**Published:** 2024-10-23

**Authors:** Rony Hirschhorn, Liad Mudrik

**Affiliations:** Sagol School of Neuroscience, Tel Aviv University, P.O. Box 39040, Tel Aviv 6997801, Israel; Sagol School of Neuroscience, Tel Aviv University, P.O. Box 39040, Tel Aviv 6997801, Israel; School of Psychological Sciences, Tel Aviv University, P.O. Box 39040, Tel Aviv 6997801, Israel; Canadian Institute for Advanced Research (CIFAR), Brain, Mind, and Consciousness Program, 661 University Ave., Suite 505, Toronto, ON M5G 1M1, Canada

**Keywords:** consciousness, contents of consciousness, natural scene perception, free report, gist, overflow, richness of perception

## Abstract

The question of the richness (or sparseness) of conscious experience has evoked ongoing debate and discussion. Claims for both richness and sparseness are supported by empirical data, yet they are often indirect, and alternative explanations have been put forward. Recently, it has been suggested that current experimental methods limit participants’ responses, thereby preventing researchers from assessing the actual richness of perception. Instead, free verbal reports were presented as a possible way to overcome this limitation. As part of this approach, a novel paradigm of freely reported words was developed using a new metric, intersubjective agreement (IA), with experimental results interpreted as capturing aspects of conscious perception. Here, we challenge the validity of freely reported words as a tool for studying the richness of conscious experience. We base our claims on two studies (each composed of three experiments), where we manipulated the richness of percepts and tested whether IA changed accordingly. Five additional control experiments were conducted to validate the experimental logic and examine alternative explanations. Our results suggest otherwise, presenting four challenges to the free verbal report paradigm: first, impoverished stimuli did not evoke lower IA scores. Second, the IA score was correlated with word frequency in English. Third, the original positive relationship between IA scores and rated confidence was not found in any of the six experiments. Fourth, a high rate of nonexisting words was found, some of which described items that matched the gist of the scene but did not appear in the image. We conclude that a metric based on freely reported words might be better explained by vocabulary conventions and gist-based reports than by capturing the richness of perception.

## Introduction

Is subjective experience rich or sparse? Although this question has puzzled researchers for centuries (Michel 2019), consciousness science has yet to agree on an answer. The topic remains highly debated, with several competing hypotheses about the richness of conscious perception (e.g. Prinz 2009, Kouider et al. 2010, Cohen and Dennett 2011, Block 2023). According to the sparseness hypothesis, cognitive functions limit what can be consciously experienced; therefore, experience is narrower than believed (Cohen et al. 2016). Conversely, the richness hypothesis suggests that experience is rich and detailed (Haun et al. 2017). In recent years, empirical evidence in support of both the sparseness hypothesis (e.g. Cohen et al. 2016, 2020) and the richness hypothesis (e.g. Vandenbroucke et al. 2012, Bronfman et al. 2014, 2019, Haun 2021, Zeleznikow-Johnston et al. 2023) has been reported. However, the evidence for both hypotheses has also been criticized, with alternative explanations for both types of results (e.g. McClelland and Bayne 2016, Phillips 2016).

Support for the richness hypothesis has been mostly based on variants of the partial report paradigm (Sperling 1960). In this method, arrays of different items are presented for brief durations. After the array disappears, participants are asked to report all the items they have seen and can typically do so only for a few of them. However, when a cue prompts participants to report only the contents of a specific row in the stimulus array (“partial report”), they can retrieve almost all items in that row (“partial report superiority”). These findings were taken by proponents of the richness hypothesis as supportive evidence, with working memory limiting access to the full, arguably rich, experience (e.g. Block 2007). According to this interpretation, the retrograde cue simply facilitates cognitive access to that rich experience. However, others challenged this view, claiming that participants may have had a conscious ambiguous experience of some of the letters, and the cue helped participants disambiguate them and report their identity (Stazicker 2011). Another alternative explanation holds that performance can be driven by nonconscious, as opposed to conscious, processing (Dehaene et al. 2006, Phillips 2016). In the Amsterdam variants of this paradigm (e.g. Landman et al. 2003, Sligte et al. 2008, Vandenbroucke et al. 2012), two arrays of shapes were presented, with the second sometimes including a changed item (e.g. presented at a different orientation). A cue marking the location of the changed item appeared at different times. Participants were better at detecting the change when the cue appeared during the interval between the displays than after the second one, which was again interpreted as evidence for the richness of subjective experience (Block 2008). Yet, this finding was also challenged; for example, Phillips (2011) claimed that participants might arguably be aware of a change without consciously perceiving each element in the two presentations. In a color diversity variant of the partial report paradigm (Bronfman et al. 2014), observers were asked to report the color diversity of letters in one of four rows, which was either cued or uncued. Reports displayed sensitivity to the color diversity of the uncued rows, at no cost to their performance at a letter recall task. The similar accuracy in the color diversity task between cued and uncued rows was interpreted as evidence for richness; this relied on the assumption that to report the diversity of colored items, participants must have retained the colors of the individual items (Block 2014). Akin to the aforementioned cases, the counterclaim again denied that the relevant contents were indeed consciously experienced: accordingly to this view, one might be able to perceive the overall color diversity of the group, without consciously experiencing each individual color (Cohen et al. 2016, Phillips 2016, Ward et al. 2016; see also Hawkins et al. 2022, reporting that color diversity is not cost-free in peripheral vision).

A similar controversy surrounds the evidence for the sparseness hypothesis. Such evidence mainly stems from attentional manipulations, like change blindness (Rensink et al. 1997, Simons et al. 2000) and inattentional blindness (IB: Simons and Chabris 1999), where participants fail to notice a salient object or a change made to a visual scene or an array. However, others claim that IB might reflect a memory failure (“inattentional amnesia”: Wolfe 1999) rather than a perceptual one: observers might have experienced the stimulus, but inattention prevented it from being encoded into memory. Another line of criticism is rooted in the controversy regarding the relationship between consciousness and attention: some researchers claim that attention and consciousness are doubly dissociated (Maier and Tsuchiya 2021), so manipulating attention does not necessarily manipulate awareness (e.g. Li et al. 2002). Under that interpretation, IB might reflect a failure to notice, rather than to consciously perceive (but see Jackson-Nielsen et al. 2017, Noah and Mangun 2020).

It is of interest that both lines of research share a common feature: they only indirectly tackle the richness debate, and they restrict responses to simple classifications (e.g. high/low: Bronfman et al. 2014; noticed/did not notice: Cohen et al. 2020; same/different: Vandenbroucke et al. 2012). These reports were claimed to be too narrow to capture the content of consciousness, creating a need for novel paradigms using free reports (Haun et al. 2017).

Recently, such a paradigm has been proposed (Chuyin et al. 2022). In that online study, participants viewed briefly presented images from a diverse stimulus set. Following each image, the observers were asked to provide five unique words describing their impressions of the image and rate their confidence in each word. Then, a novel metric called “intersubjective agreement” (IA) was introduced, quantifying the uniqueness of the reported words with respect to the image they described. The authors focused on the “word IA” metric, which depicts the relationship between the frequency of a word describing a particular image, as opposed to its frequency describing all the other images in the dataset. This metric was reported to be high, even for briefly presented stimuli (67 ms). The authors claimed that such high IA demonstrates that even a brief glance at an image allows a highly detailed and specific conscious experience, such that participants perceive not only the gist of the scene, or its general theme (Oliva 2005), but also many of its details (e.g. small objects). This led to the conclusion that the IA measure can be used to capture the content of conscious perception (Chuyin et al. 2022).

Here, we revisit this conclusion and ask whether free verbal reports can indeed be used to capture the richness of perception. We replicated the original experiment and then degraded the stimuli in two ways, in order to uniquely test disconfirmatory predictions (Firestone and Scholl 2016). Specifically, we asked if the measure would yield differential results, under the assumption that these changes affect the content of perception. This series of three experiments was first conducted in an exploratory study and then replicated in a preregistered study.

**Figure 1:**
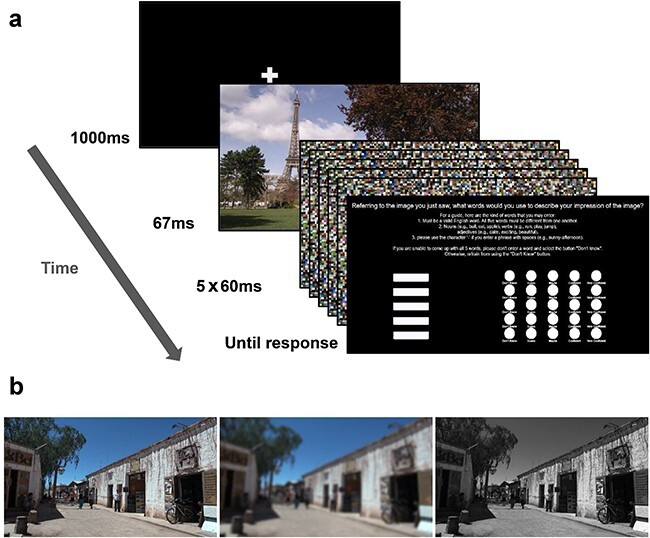
(a) Procedure for all three experiments in the exploratory and preregistered studies. Each trial began with a fixation, followed by a 67-ms presentation of the target stimulus. Then, the stimulus was backward-masked with five successive masks, each lasting for 60 ms. Finally, a response screen appeared, prompting participants to provide five words describing their experience of the image and rate their confidence in each word. The response screen had no time limit. The panel was adapted from the original work (Chuyin et al. 2022). (b) An example for the three stimulus versions: intact (left), blurred (middle), and black and white (right).

In these studies, stimuli were presented for the shortest stimulus duration used in the original work (67 ms; Chuyin et al. 2022, see Fig. 1), on which the critical claims were made. The first experiment (“intact”) was identical to the original paradigm. In the second experiment (“blurred”), we repeated the first experiment but with blurred versions of the images. We expected blurred stimuli to evoke less rich and detailed percepts (e.g. Prinz 2009, French 2015) as they diminish the fine details of the scene, thereby reducing the ability to bind contours and detect objects (Oliva 2005). This assumption was tested and supported in two control experiments (manipulation check; see the “Materials and methods” section). In the third experiment (“black and white”), all images appeared in grayscale, thereby removing one aspect of the original experience (color), making it less rich. We reasoned that if the word IA measure indeed captures perceptual experience, it should decrease in both the blurred and black and white experiments, compared to the intact experiment. This reasoning allowed us to test the construct validity of the IA metric (i.e. whether it captures the phenomenon it is meant to measure; Cronbach and Meehl 1955).

In addition to the main studies, we conducted five control experiments, to better substantiate our conclusions. Two control experiments tested the underlying rationale of the main experiments, according to which our experimental manipulation indeed degraded the experienced content. As this seems evident for the color manipulation, we focused on the blurriness manipulation and asked whether blurred stimuli were less rich than intact ones (manipulation check). Three *post hoc* control experiments were conducted to rule out alternative explanations to the results we found in the main study (a direct replication of the original work (Chuyin et al. 2022) and two long-exposure experiments).

## Materials and methods

### Main studies

Both the exploratory study and the preregistered one included three online experiments: the first one presenting the stimuli used in the original work (Chuyin et al. 2022; “intact”), the second presenting a blurred version of the stimuli (“blurred”), and the third presenting a desaturated version of the stimuli (“black and white”). We first detail the shared attributes of all six experiments (three for each study). Then, in each section, we specify the unique characteristics of each study. Additional control experiments are described in a separate section further.

#### Participants

Participants in all six experiments were recruited using the Prolific platform, based on the following requirements: age 18–35 years, with English as their first language (as verified by Prolific), a 95% approval rate or higher (in the exploratory study experiments: 90%), and participating via a desktop computer (rather than an iPad or phone). Participants read the instructions and provided their informed consent in Prolific. Then, they were automatically transferred into the experimental platform (see the “Apparatus” section).

##### Exploratory study

###### Intact

A total of 209 participants completed the online experiment (106 females, 102 males, and 1 unreported, aged 19–35 years: *M* = 27.17, SD = 4.58). Participants were paid a rate of 7.52£/h (duration *M* = 24.56 minutes, SD = 13.91). One additional participant did not complete the experiment and was removed from further analysis.

###### Blurred

A total of 218 participants completed the online experiment (109 females, 108 males, and one unreported, aged 19–35 years: *M* = 26.88, SD = 4.76). Participants were paid a rate of 7.52£/h (duration *M* = 17.64 minutes, SD = 10.82). Following the exclusion criteria in the original study, four participants were removed from the analysis due to the stimulus balancing method (requiring ten respondents per image; see “IA calculation” section). Therefore, the final sample included 214 participants. Three additional participants were not analyzed as they did not complete the experiment.

###### Black and white

A total of 203 participants completed the online experiment (102 females and 101 males, aged 18–35 years: *M* = 27.10, SD = 4.76). Here and in later experiments, participants were paid a rate of 9£/h (duration *M* = 20.24 min, SD = 10.02). Ten additional participants did not complete the experiment and were removed from further analysis.

##### Preregistered study

###### Intact

A total of 206 participants completed the online experiment (103 females and 103 males, aged 18–35 years: *M* = 27.73, SD = 4.72). The average duration was 22.50 min (SD = 12.10). Four additional participants were excluded since they did not complete the experiment.

###### Blurred

A total of 212 participants completed the online experiment (107 females and 105 males, aged 18–35 years: *M* = 27.36, SD = 4.39). The average duration was 19.73 min (SD = 17.24). Four additional participants did not complete the experiment and were removed from further analysis.

###### Black and white

A total of 211 participants completed the online experiment (104 females and 107 males, aged 18–35 years: *M* = 27.57, SD = 4.71). The average duration was 19.12 min (SD = 10.36). Eight additional participants did not complete the experiment.

#### Apparatus

The experiments were programmed in Python and Javascript using Psychopy (Peirce et al. 2019) and were executed online via Pavlovia (an online platform for Psychopy-based experiments). As the original paradigm and analyses required ten respondents per stimulus (Chuyin et al. 2022), stimulus presentation sequences were generated in advance for each experiment to ensure that each image would be presented to ten different participants.

Because online experiments are more prone to timing inaccuracies due to differences between participant setups (Bridges et al. 2020), special care has been taken to mitigate these discrepancies and minimize the potential variance of timings: all the stimulus image files and their order (i.e. the stimulus presentation sequences) were loaded in advance once participants entered the platform. The experiments accordingly began only once all the experimental software materials were loaded. The experiments were limited to running on desktop computers and were run in full-screen mode.

#### Stimuli

The stimulus set was taken from the original work (Chuyin et al. 2022). The same three images were used as practice images. Out of the 412 experimental images, and following Supplementary Fig. 2 in the original work, we removed duplicate images such that in the exploratory study, 393 stimuli were used. Prior to the preregistered study, we identified five additional pairs of images as duplicates and removed them from the preregistered stimulus set, yielding 388 unique images. The five masking images were taken from the original work.

In both studies, the practice, masking, and experimental stimuli have been modified in the blurred and black and white experiments: in the blurred version, all images were blurred using Photoshop’s Gaussian blur with a 10-pixel radius, and in the black and white version, all images were grayscaled (Adobe Inc 2019).

#### Procedure

After providing their informed consent in Prolific, participants were rerouted to the Pavlovia platform on which the experiments were run. Then, participants saw a series of self-paced instruction screens (presented for at least 5 s each) explaining that during the experiment, they would see briefly presented images and would be asked to write and rate five unique English words (nouns, verbs, and adjectives) describing their impression of each image. Next, participants continued to three practice trials.

All experiments consisted of 21 trials; each participant’s trial sequence was loaded from predefined lists based on the order in which they entered the experiment platform. Figure 1(a) outlines the structure of a single trial. During each trial, a stimulus image was presented for 67 ms, followed by five successive masking images, each presented for 60 ms. Then, a response screen appeared. Akin to the original work, the screen included instructions about how participants are asked to respond and five text boxes with five corresponding confidence scores (1: “Don’t Know,” 2: “Guess,” 3: “Maybe,” 4: “Confident,” and 5: “Very Confident”). Observers were encouraged to write five words. If they could not come up with five words, they were instructed to leave the text box empty and rate their response as “Don’t Know” (differing from the original work, where participants were instructed to insert arbitrary words in such a case and rate them as “Don’t Know”: Chuyin et al. 2022; see the “Direct replication” section for a control experiment, replicating the original work with its exact instructions). After 21 trials, participants were thanked for their participation and automatically redirected out of the experiment platform.

#### Data preprocessing

Our data preprocessing mimics the original work’s pipeline. As per the original work, in cases where more than ten participants responded to the same image, only the first ten responses were taken for subsequent analyses. Words were processed similarly to the original work (e.g. response words were converted to lowercase, and spaces were converted to hyphens). We also converted digits to verbal descriptions of numbers (e.g. “2” was converted to “two”).

After parsing the response words, we followed the original procedure using Python. A Python spellchecking package (pyspellchecker; version 0.7.2) was run on all parsed response words. Then, the results were exported to a file containing words found to be misspelled by the spell-checker, with their corresponding suggested corrections. A manual check was then performed to determine whether to accept each correction (e.g. “holiaday” to “holiday” was deemed an acceptable correction, while “red-dot” to “reddit” was not), and the dataset was updated accordingly. Then, word lemmatization was performed using an industrial-strength natural language processing Python package (spaCy: Honnibal and Montani 2017, version 3.6.0). Once again, the lemmatization results were exported to a file containing the original and lemmatized versions, and a manual inspection was performed to approve the lemmatization outputs (e.g. “loving” to “love” was approved, while “t-shirt” to “t” was not). Finally, we verified that within a given trial, all response words of a single person were unique (repetitions, if found, were removed). Other than the two manual steps that followed the original work (spelling and lemmatization approvals), the rest were all automatically performed using Python software.

#### IA calculation

The IA metric calculation was also identical to the original work. In each experiment, the IA analysis was performed per image (“target image”), per word (“target word”). Therefore, within each experiment, the word IA is a number associated with a word–image pair (e.g. the word “rainbow” can potentially have 388 different IA scores, one per image in the experiment database).

The first step was to create a response matrix for each image in the dataset. Preprocessed responses for the image from all ten observers were aggregated into a matrix containing ten rows (one per participant) and five columns (one per word). Because multiple participants can provide the same word, each word can appear between one and ten times in the matrix. Crucially, akin to the original work, a word reported only once per image was considered a “rarely reported word” (as it was reported by one observer only) and removed from the IA analysis.

The word IA calculation was then done iteratively within the image: for each row (participant), we analyzed each target word. Notably, following the original work, the analysis was performed for each unique combination of word and row. Thus, as the target word appeared in at least one other row, the IA analysis was conducted again (as the index of the target word’s row plays a role in the calculation; see Chuyin et al. 2022 and the “IA calculation” section below). We calculated the “within-image” ratio by counting the number of additional occurrences of the target word in the target image matrix and dividing it by nine (the maximal possible number of repetitions).

The next step was to calculate the “between-image” ratios for the target word in this specific row, in order to generate a distribution of these ratios for that combination. This was done by counting, for all the other images, the number of times the target word appeared in their matrix. To that end, the corresponding row where the target word appeared in the target image was removed, and the number of occurrences of that word in all other rows was divided by nine.

To generate the distribution, we defined ten bins, each representing one possible between-image ratio (0/9 to 9/9), and counted the number of images in each bin. Then, we turned the image counts into cumulative counts (see Fig. 2 in Chuyin et al. 2022), for both the within- and the between-image ratios.

**Figure 2:**
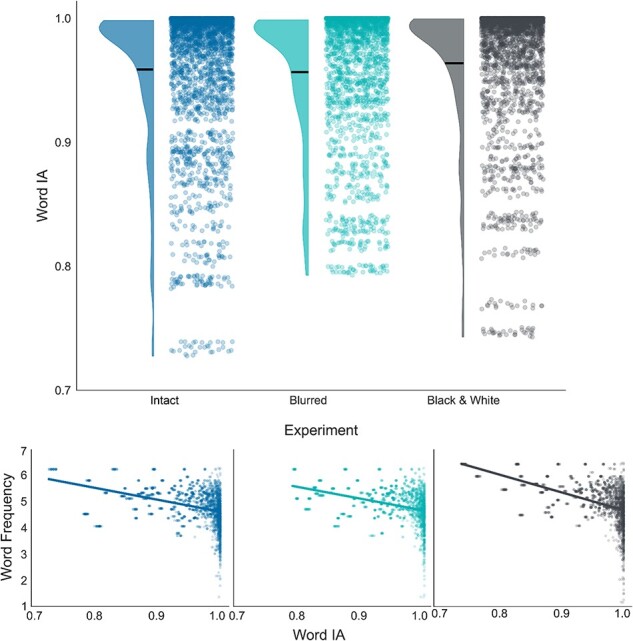
Exploratory study results. Notably, both Zipf frequency (word ranks) and word IA (specificity scores) are described in arbitrary units. (Top) A raincloud plot showing the distribution of word IA scores across experiments. The dots illustrate individual words, while the density curves denote their distribution. Horizontal lines mark the means. (Bottom) Scatter plots showing the correlation between words’ IA (*x*-axis) and their frequency in English (*y*-axis). The scatter plots are separated by experiments, with colors matching the top plot (left: intact, middle: blurred, right: black and white).

These ratios were used to construct a receiver operating characteristic curve, where the cumulative within-image ratio is considered the “true-positive rate,” as it represents the frequency of the target word in the target image. The between-image cumulative ratio is considered the “false-positive rate,” as it represents the frequency of the target word in nontarget images.

Finally, the area under the curve (AUC) was calculated. This process was repeated for all rows in which the target word appeared in that image, and the final word IA score was the average of all AUCs across rows.

#### Statistical analysis

Statistical analyses were all performed using JASP software (JASP Team 2022; version 0.17.2.1), first on the results of the exploratory study and then on the preregistered study.

To test whether word IA differs across stimulus versions, we performed a between-participant Bayesian one-way analysis of variance (ANOVA) with experiment as the fixed effect (intact, blurred, black and white) and word IA as the dependent variable.

To test the correlation between words’ IA score and their frequency in the English vocabulary, we performed a Bayesian Pearson correlation between these variables separately for each experiment. Word frequency was extracted using Python’s wordfreq package (version: 3.0.3), and the frequency score used was Zipf frequency: The Zipf frequency of a word is the base-10 logarithm of the number of times it appears per billion words (Brysbaert and New 2009; see more at pypi.org/project/wordfreq/).

To test the correlation between words’ IA and their mean confidence level, we performed a Bayesian Pearson correlation between these variables, separately for each experiment.

**Figure 3:**
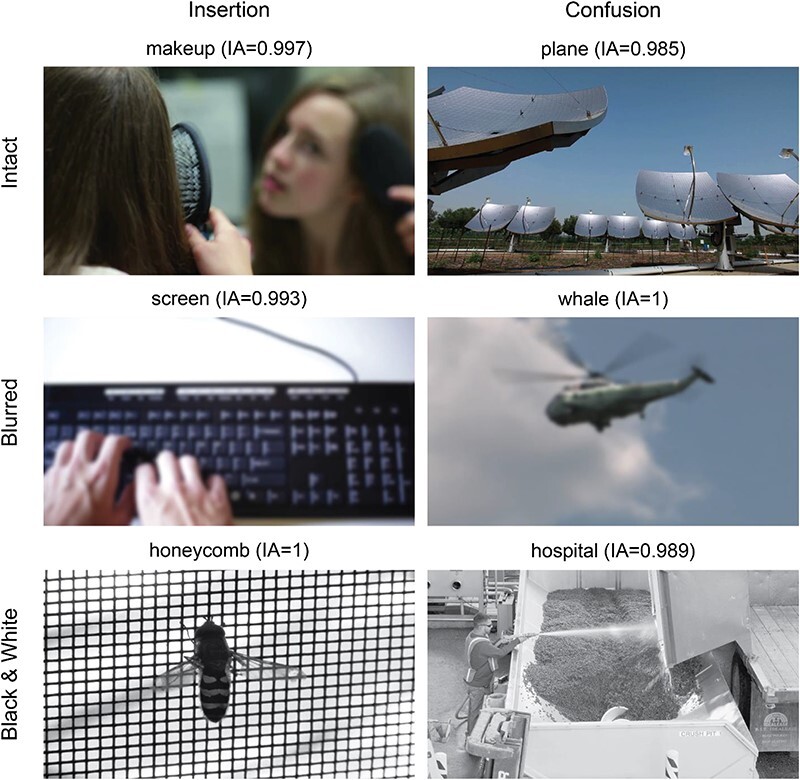
Examples of nonexisting words of the insertion (left) and confusion (right) types in the exploratory study. Each row is an experiment (top: intact stimuli, middle: blurred stimuli, bottom: black and white). Above each image is the word provided for it and its respective word IA score.

Finally, we examined how many of the words described items that did not appear in the image (i.e. nonexisting words), using only nonrare words (i.e. only words with IA scores), and characterized them. Notably, the purpose of this exploratory analysis was not to find an alternative to the word IA metric. Rather, we examined the content of those words to better understand what the word IA measure actually captures. We accordingly identified four types of nonexisting words (see Figs 3 and 7 in the “Results” section for example): (i) “conceptual”: words that convey a sentiment or some abstract description of the image, rather than a concrete item/attribute that appears in it (e.g. “cute,” “intricate,” “boring,” and “romantic”); (ii) “insertion”: words describing concrete items that are congruent with the scene’s gist, but do not appear in the image (e.g. “horse” and “book” when these are not present in the image); (iii) “confusion”: words that do not match the scene or its gist, but seem to describe an “alternative” gist that is congruent with the features of the scene, such that participants most likely perceived this alternative gist, although it does not appear in the image (e.g. “whale” in response to an image of the sky, suggesting that observers mistook the sky as an ocean; see Fig. 3); and (iv) “unrelated”: a word that does not describe something that exists in the image and does not belong to any of the abovementioned categories, having no connection whatsoever to the image, to our best judgment.

This classification process was done manually and separately for each experiment (while “red” can be an existing word in an image in the intact experiment, it does not exist for the same image in the black and white experiment). To minimize subjective judgment, the process was done by both authors, and disagreements were resolved by discussion. We took a lenient approach when classifying the words, such that even words that did not clearly appear in the image, but could be ascribed to it (e.g. the word “sunny” when no sun or rays of light were depicted), were considered to be existing in the image and were not counted as “nonexisting.”

For each image, we counted the total number of words with word IA scores. We calculated the proportion of existing and nonexisting words (of all types) by dividing the number of words in each category by the number of words with IA scores in that image. Then, we examined the relationship between words’ categories and their mean confidence levels using a Bayesian ANOVA with confidence as the dependent variable and category as a fixed effect.

### Control experiments

#### Manipulation check

The results of our studies hinge upon the assumption that blurring the images or changing them to grayscale reduces the richness of experiencing them. While this seems almost self-evident for the grayscale images, as colors influence the vividness of one’s perception (Cornoldi et al. 1991), one might claim that this is not the case for blurring or that our specific blurring protocol was not strong enough to reduce the perceived richness. Two control experiments were performed to test this claim.

##### Participants

Participants in both experiments were recruited using Prolific in an identical manner to the exploratory study and paid at a rate of 9£/h. Twenty participants (10 females) completed the first control experiment (aged 21–34 years: *M* = 27.5, SD = 4.37), which lasted 8.92 min on average (SD = 6.97), and the other 20 (11 females) completed the second control experiment (aged 21–34 years: *M* = 26.90, SD = 3.57), which lasted 15.44 min (SD = 13.01).

##### Procedure, stimuli, and apparatus

The apparatus and stimuli were identical to those used in both studies, with only one practice pair. The first control experiment (“Control Experiment A”) included 20 trials, in which pairs of intact and blurred versions of the same image were presented. Participants were asked to report which was richer. The location (left, right) of the intact and blurred versions was counterbalanced, and the order was random, with every pair presented only once.

Akin to the main studies, in each trial, the stimulus pair was presented for 67 ms, followed by five successive pairs of masks, presented for 60 ms—covering each image separately. A response screen then prompted participants to indicate which version was richer, and they responded using their keyboard arrow keys (left and right).

Notably, in the first control experiment, participants’ richness judgments might have been biased given that the images were presented simultaneously, potentially hinting that intact images should be rated as richer. Also, this experiment did not allow us to quantify the difference in perceived richness. To mitigate both concerns, in the second experiment (“Control Experiment B”), each image was presented in isolation, and participants were asked to rate how rich was their experience of a single image on a continuous scale ranging between “Very poor experience” (coded 0) and “Very rich and detailed experience” (coded 1), by moving the computer mouse to set a location of a marker on the scale (the marker was invisible until participants chose the desired location on the scale, to not bias their response in any direction).

The experiment consisted of 60 test trials, 30 depicting blurred images and 30 depicting intact images, which were not paired (i.e. no participant saw both the intact and blurred versions of the same image).

#### Direct replication

One of the key results of the original study is the correlation between word IA and confidence rating (Chuyin et al. 2022). As we did not find it in any of the six main experiments (exploratory and preregistered), it is important to rule out that this failure to replicate stems from the slight change in instructions concerning the lowest confidence score (1: “Don’t Know”). Thus, in this control experiment, we used the original instructions, such that observers were asked to insert an arbitrary word if they could not come up with five words (and rate it as “Don’t Know”). The procedure, stimuli, and apparatus were otherwise identical to those in the preregistered intact experiment.

##### Participants

Participant recruitment was identical to the preregistered intact experiment. A total of 232 participants completed the online experiment (116 females, aged 18–35 years: *M* = 28.38, SD = 4.63). The average duration was 20.03 min (SD = 10.39). No additional participants were removed for not completing the experiment.

#### Long exposure

The original work has emphasized the importance of finding high word IAs in briefly presented images (67 ms). In our main study, we did not find the metric to be sensitive to stimulus manipulations. However, this insensitivity might stem from the short presentation duration, and one might still argue that the IA measure can better capture experience when the stimuli are presented for longer. To test this hypothesis, we repeated the preregistered intact and black and white experiments, with images presented for 267 ms (the longest duration in the original work). The procedure, stimuli, apparatus, and participant recruitment were otherwise identical to the preregistered experiments.

##### Participants

###### Intact

A total of 201 participants completed the online experiment (100 females and 101 males, aged 18–35 years: *M* = 27.89, SD = 4.56). The average duration was 19.99 min (SD = 9.73). No additional participants were excluded for not completing the experiment.

###### Black and White

A total of 205 participants completed the online experiment (102 females and 103 males, aged 18–35 years: *M* = 27.75, SD = 4.47). The average duration was 19.82 min (SD = 10.34). Three additional participants were excluded since they did not complete the experiment.

## Results

### Exploratory study

Overall, the experiment (i.e. the manipulation of richness) was found to significantly affect word IA [*F*(2, 6833) = 9.720, *P* < .001, BF_10_ = 27.50; Fig. 2, top]. *Post hoc* analyses revealed that the black and white word IAs (*M* = 0.964, SD = 0.05) were actually slightly higher than both the blurred condition [*M* = 0.957, SD = 0.05; *t*(6833) = 4.227, *P* < .001, 95% confidence interval (CI) = (0.003, 0.011), BF_10_ = 360.61] and the intact one [*M* = 0.959, SD = 0.06; *t*(6833) = 3.204, *P* = .004, 95% CI = (0.001, 0.009), BF_10_ = 5.14]. Notably, no difference was found between the intact and blurred versions [*t*(6833) = −1.249, *P* = .635, 95% CI = (−0.006, 0.002), BF_10_ = 0.07].

This might suggest that IA captures things other than conscious perception. For example, this measure might reflect lexical regularities, independent of the experienced contents. In this case, IA scores should correlate with other vocabulary-based metrics of words. To test that, we examined the relationship between words’ IA scores and their frequency in the English vocabulary. Indeed, across all three experiments, a negative correlation was found between word IA and frequency [intact: *r*(2569) = −0.374, *P* < .001, 95% CI = (−0.407, −0.341), BF_10_ = 2.86 × 10^82^; blurred: *r*(2013) = −0.369, *P* < 0.001, 95% CI = (−0.406, −0.330), BF_10_ = 1.36 × 10^62^; black and white: *r*(2254) = −0.471, *P* < 0.001, 95% CI = (−0.502, −0.438), BF_10_ = 6.49 × 10^120^; Fig. 2, bottom]. That is, the more specific a word was found to be for a particular image, the more unique the word was in English in general, in line with the hypothesis that the measure possibly reflects lexical regularities.

Furthermore, the positive correlation between word IA and confidence found in the original study (Chuyin et al. 2022) and held by the authors to validate the measure as indicative of conscious perception, was not replicated in any of the three experiments. In two out of the three experiments, no significant correlation was found: the intact [*r*(2569) = −0.021, *P* = .280, 95% CI = (−0.060, 0.017), BF_10_ = 0.04] and the blurred [*r*(2013) = −0.041, *P* = 0.067, 95% CI = (−0.084, 0.003), BF_10_ = 0.15]. In the black and white experiment, the correlation was in the opposite direction of what was originally found [*r*(2254) = −0.066, *P* = .002, 95% CI = (−0.107, −0.025), BF_10_ = 3.81].

**Figure 4:**
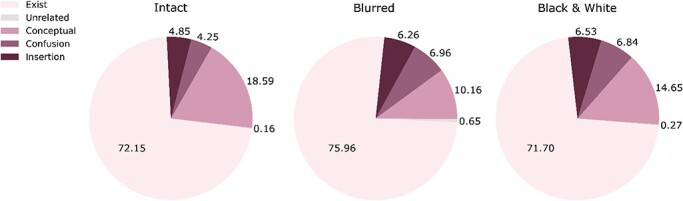
Average proportion of words of each category type per image (standard deviations: intact experiment: exist = 18.77, conceptual = 15.76, insertion = 9.21, confusion = 9.98, unrelated = 1.82; blurred experiment: exist = 21.34, conceptual = 14.33, insertion = 13.12, confusion = 14.39, unrelated = 5.25; black and white experiment: exist = 21.85, conceptual = 15.53, insertion = 13.92, confusion = 14.34, unrelated = 2.53). Each pie depicts the results of a separate experiment in the exploratory study (left: intact stimuli, middle: blurred stimuli, right: black and white). “Exist” words describe things that existed in the image; “Conceptual” words convey sentiment, adjectives, or any nonconcrete, abstract words related to the image; “Insertion” words describe items that do not appear in the image, but are compatible with its gist; “Confusion” words do not exist in the image nor compatible with its true gist, yet are compatible with an alternative (wrong) gist of the image; “Unrelated” words are entirely unrelated to the image, not falling under any of the previous categories. Here and in Figs 5–9s, all words included in this classification have an IA score.

Another possible nonperceptual explanation for the IA results is that participants were not able to extract enough information from the images, relying on gist perception to provide words describing items that are compatible with the presented images (even if they did not actually see them). If this is true, some of the words provided for each image are expected to be of the “insertion” nonexistent type (i.e. words that describe items that do not appear in the scene, but are compatible with its gist; Fig. 3, left). Indeed, across all three experiments, such insertion words were found (Fig. 4).

To further examine if our classifications captured different types of responses, we compared the confidence ratings of words in each category. If our existence judgments were not aligned with the reported words (i.e. if the words did not fall into the categories we identified), we would expect no relationship between the rated confidence and the existence tagging of those words.

**Figure 5:**
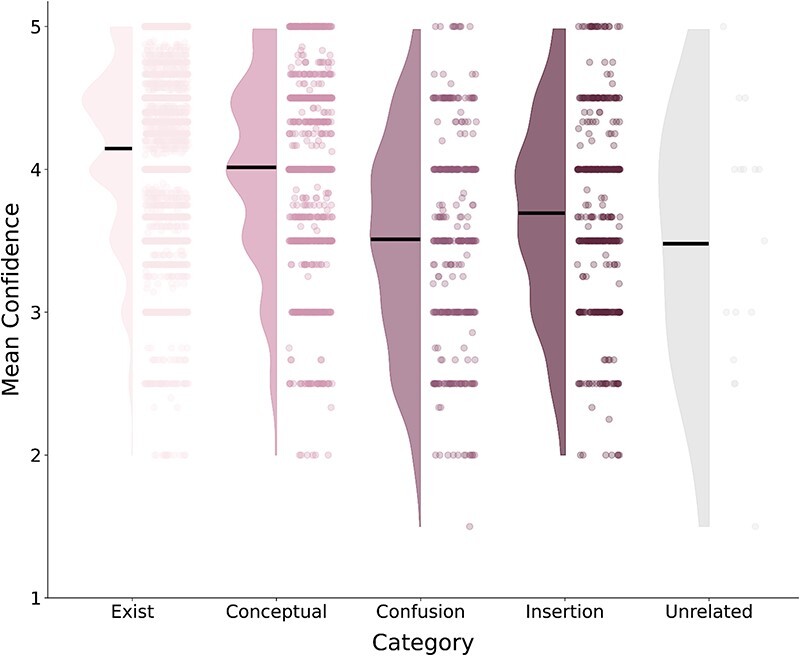
A raincloud plot showing the distribution of confidence ratings (*y*-axis) across word category types (*x*-axis) in the exploratory study. The dots illustrate individual words, while the colored density curves denote their distribution. Horizontal lines mark the means.

Contrary to those expectations, in the exploratory study (all three experiments together), word categories did differ in their confidence ratings [*F*(4, 6855) = 139.53, *P* < .001, BF_10_ = 3.83 × 10^111^] such that existing words (*M* = 4.15, SD = 0.58) had higher confidence compared to each of the nonexisting word types (conceptual: *M* = 4.01, SD = 0.65; confusion: *M* = 3.51, SD = 0.73; insertion: *M* = 3.69, SD = 0.70; unrelated: *M* = 3.48, SD = 0.93; Fig. 5 and Table 1). Thus, participants were less confident in their reports when providing nonexisting words.

**Table 1.** Results of post hoc comparisons of the Bayesian ANOVA of words’ mean confidence per category (exist, conceptual, insertion, confusion, and unrelated) in the exploratory study.

		Mean difference	95% CI for mean difference					
	Mean difference		Lower	Upper	SE	*t*	*P*_bonf_	BF_10,U_
Exist	Confusion	0.64	0.55	0.73	0.03	19.248	<.001^***^	2.15 × 10^+80^
	Unrelated	0.67	0.25	1.08	0.15	4.359	<.001^***^	2630.34
	Insertion	0.45	0.37	0.54	0.03	14.140	<.001^***^	5.55 × 10^+43^
	Conceptual	0.13	0.08	0.19	0.02	6.473	<.001^***^	1.14 × 10^+8^
Conceptual	Confusion	0.50	0.40	0.60	0.04	13.698	<.001^***^	4.25 × 10^+30^
	Unrelated	0.54	0.12	0.95	0.15	3.479	.005^**^	24.25
	Insertion	0.32	0.22	0.42	0.04	8.942	<.001^***^	1.16 × 10^+13^
Confusion	Unrelated	0.03	−0.40	0.46	0.16	0.197	1	0.26
	Insertion	−0.18	−0.30	−0.06	0.04	−4.135	<.001^***^	37.38
Unrelated	Insertion	−0.21	−0.64	0.21	0.16	−1.375	1	0.47

All words included in this classification have an IA score. Abbreviations: *P*_bonf,_ Bonferroni-corrected *P*-value (** *P* ≤ .01, *** *P* ≤ .001); SE, standard error.

### Preregistered study

**Figure 6:**
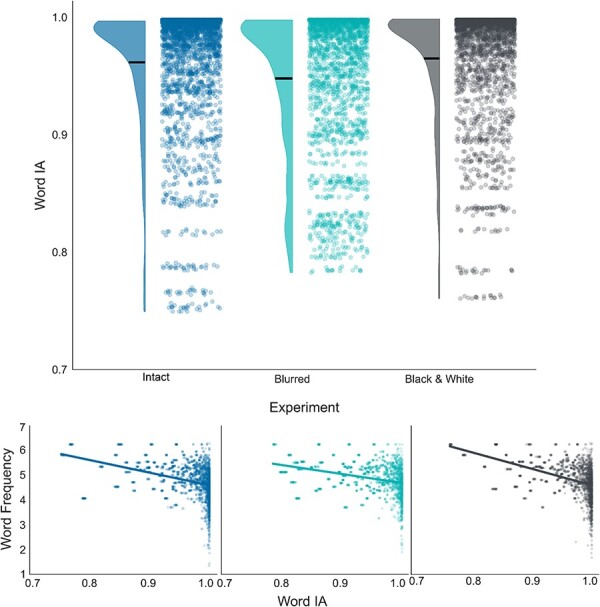
Preregistered study results. (Top) A raincloud plot showing the distribution of word IA scores across experiments. The dots illustrate individual words, while the density curves denote their distribution. Horizontal lines mark the means. (Bottom) Scatter plots showing the correlation between words’ IA (*x*-axis) and their frequency in English (*y*-axis). The scatter plots are separated by experiments, with colors matching the top plot (left: intact, middle: blurred, right: black and white).

Once again, the experiment (i.e. richness manipulation) was found to significantly affect IA [*F*(2, 6785) = 62.180, *P* < 0.001, BF_10_ = 7.58 × 10^23^; Fig. 6, top). However, the patterns of results were somewhat different from those in the exploratory study: word IA in the black and white experiment (*M* = 0.967, SD = 0.05) was again higher than in the blurred experiment [*M* = 0.950, SD = 0.06; *t*(6785) = 10.514, *P* < .001, 95% CI = (0.013, 0.021), BF_10_ = 5.93 × 10^21^], but not significantly higher than in the intact experiment [*M* = 0.964, SD = 0.05, *t*(6785) = 2.147, *P* = .096, 95% CI = (−3.02 × 10^−4^, 0.007), BF_10_ = 0.42]. Thus, in both studies, color degradation did not lead to the decrease in word IA scores that would have been expected if this measure had indeed captured the richness of perception. However, unlike in the exploratory study, word IA in the blurred experiment was found to be lower than in the intact experiment [*t*(6785) = −8.792, *P* < .001, 95% CI = (−0.017, −0.010), BF_10_ = 6.03 × 10^13^].

All other results were fully replicated in this study: first, the negative correlation between words’ IA scores and frequency in English was found across all three experiments [Fig. 6 (bottom): intact: *r*(2561) = −0.368, *P* < .001, 95% CI = (−0.401, −0.334), BF_10_ = 2.20 × 10^79^; blurred: *r*(2031) = −0.325, *P* < .001, 95% CI = (−0.364, −0.286), BF_10_ = 5.14 × 10^47^; black and white: *r*(2196) = −0.443, *P* < 0.001, 95% CI = (−0.476, −0.409), BF_10_ = 2.89 × 10^102^]. Second, the positive relationship originally reported between word IA and the confidence level was again not found in any of the experiments [intact: *r*(2561) = −0.015, *P* = .434, 95% CI = (−0.054, 0.023), BF_10_ = 0.03; blurred: *r*(2031) = 0.013, *P* = .550, 95% CI = (−0.030, 0.057), BF_10_ = 0.03; black and white: *r*(2196) = −0.027, *P* = .198, 95% CI = (−0.069, 0.014), BF_10_ = 0.06].

**Figure 7:**
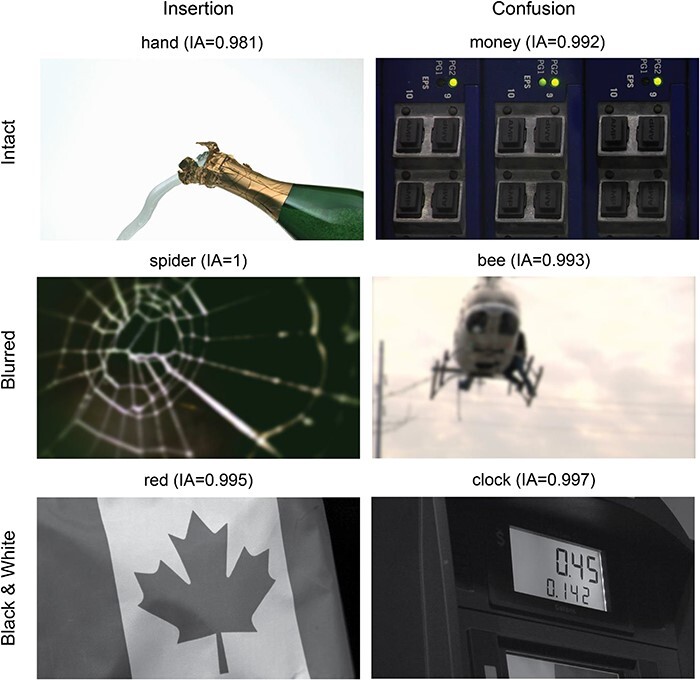
Examples of nonexisting words of the insertion (left) and confusion (right) types in the preregistered study. Each row is an experiment (top: intact stimuli, middle: blurred stimuli, bottom: black and white). Above each image is the word provided for it and its respective word IA score.

**Figure 8:**
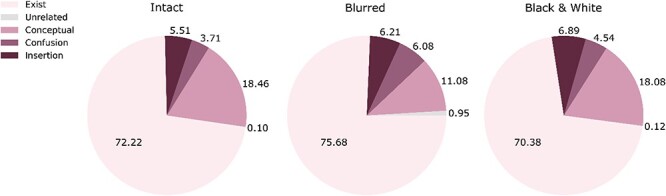
Average proportion of words of each category type per image (standard deviations: intact experiment: exist = 17.99, conceptual = 15.18, insertion = 9.90, confusion = 9.39, unrelated = 1.09; blurred experiment: exist = 21.99, conceptual = 14.34, insertion = 13.48, confusion = 14.51, unrelated = 6.12; black and white experiment: exist = 22.33, conceptual = 17.71, insertion = 13.10, confusion = 12.11, unrelated = 1.64). Each pie depicts the results of a separate experiment in the preregistered study. The same conventions as in Fig. 4 are used here.

Third, the proportions of existing and nonexisting words per image were similar to those of the exploratory study. Specifically, the percentage of nonexisting “insertion” words per image was again consistent across all three experiments and of the same magnitude found in the exploratory studies (see Fig. 7 for examples and Fig. 8 for the proportions).

**Figure 9:**
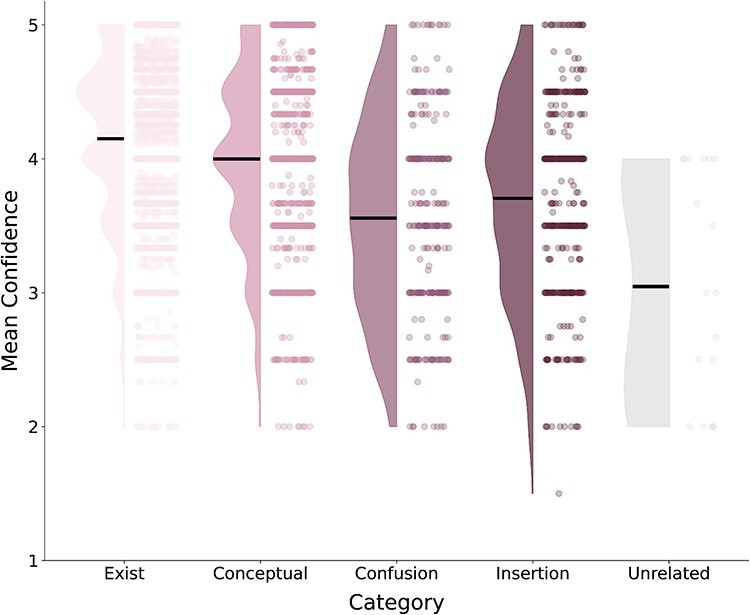
A raincloud plot showing the distribution of confidence ratings (*y*-axis) across word category types (*x*-axis) in the preregistered study. The dots illustrate individual words, while the colored density curves denote their distribution. Horizontal lines mark the means.

Fourth, the relationship between these classifications and confidence ratings was replicated as well [*F*(4, 6783) = 120.586, *P* < .001, BF_10_ = 4.78 × 10^95^], with existing words (*M* = 4.15, SD = 0.61) having higher confidence ratings compared to all types of nonexisting ones (conceptual: *M* = 4.00, SD = 0.65; confusion: *M* = 3.56, SD = 0.73; insertion: *M* = 3.70, SD = 0.72; unrelated: *M* = 3.05, SD = 0.85; Fig. 9 and Table 2).

### Control experiments

#### Manipulation check

##### Control experiment A

For each participant, we calculated the percentage of trials where they selected the intact version as richer than the blurred one. A Bayesian one-sample *t*-test was performed to test whether the percentage differed from chance level (50%). An additional Bayesian paired samples *t*-test was used to test whether participants preferred a side when selecting which image was richer (left, right).

As expected, participants selected the intact version of the image as richer than the blurred one on average 74.5% of the time [SD = 13.66; *t*(19) = 8.021, *P* < .001, 95% CI = (18.11, 30.89), BF_10_ = 95 836.62]. In addition, there was no effect of presentation side on richness preference [*t*(19) = 0.474, *P* = 0.64, 95% CI = (−10.25, 16.25), BF_10_ = 0.26, BF_01_ = 3.89].

##### Control experiment B

Participants’ richness judgments were compared in three ways. First, we conducted a participant-based analysis and used a Bayesian paired samples *t*-test to compare the average ratings participants gave to intact vs. blurred images. Second, we focused only on the first trial to minimize the effect of participants associating “rich” with “nonblurred” throughout the experiment. An independent samples *t*-test was accordingly performed to test whether on the first trial, intact images were rated differently than blurred ones. Third, we conducted an image-based analysis and compared richness judgments between two versions of the same image (intact, blurred) using a Bayesian paired samples *t*-test.

**Figure 10:**
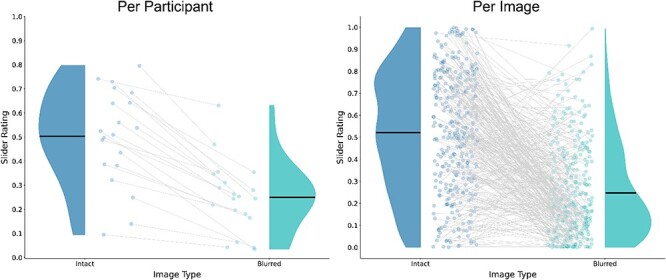
Results of control experiment B (across all trials). (Left) Results per participant. Each dot represents the average slider rating of a single participant across all trials of the same image type (intact, blurred). Violins are the distributions across participants. Gray lines connect between two trial groups of the same participant. As seen by these lines, each participant consistently rated blurred images (on the right) lower than intact images (on the left). (Right) Results per image. Each dot represents the average slider rating of a single image across all participants who viewed it. Violins are the distributions across images. Gray lines connect between two versions of the same image.

Overall, participants gave higher richness ratings to the intact images (*M* = 0.50, SD = 0.20) compared to blurred ones [*M* = 0.25, SD = 0.15; *t*(19) = 9.322, *P* < .001, 95% CI = (0.20, 0.31), BF_10_ = 805 258.13; Fig. 10, left]. Importantly, during the first experimental trial, where participants were less prone to be biased by understanding the manipulation, intact images (*M* = 0.59, SD = 0.21) were still rated much higher than blurred ones (*M* = 0.17, SD = 0.13) on the richness of experience scale [*t*(18) = 5.414, *P* < .001, 95% CI = (0.25, 0.58), BF_10_ = 443.93]. Furthermore, the image-based analysis revealed that the intact versions were given higher richness ratings (*M* = 0.52, SD = 0.27) compared to their blurred counterparts [*M* = 0.25, SD = 0.21; *t*(299) = 14.42, *P* < .001, 95% CI = (0.24, 0.31), BF_10_ = 6.54 × 10^32^].

**Table 2.** Results of *post hoc* comparisons of the Bayesian ANOVA of words’ mean confidence per category in the preregistered study.

		Mean difference	95% CI for mean difference					
	Mean difference		Lower	Upper	SE	*t*	*P*_bonf_	BF_10,U_
Exist	Confusion	0.59	0.49	0.70	0.04	15.662	<.001^***^	5.65 × 10^+52^
	Unrelated	1.10	0.70	1.51	0.15	7.438	<.001^***^	2.14 × 10^+11^
	Insertion	0.45	0.36	0.53	0.03	14.124	<.001^***^	3.78 × 10^+42^
	Conceptual	0.15	0.10	0.21	0.02	7.471	<.001^***^	1.24 × 10^+11^
Conceptual	Confusion	0.44	0.33	0.55	0.04	10.780	<.001^***^	2.31 × 10^+20^
	Unrelated	0.95	0.55	1.36	0.15	6.383	<.001^***^	6.83 × 10^+6^
	Insertion	0.29	0.20	0.39	0.04	8.350	<.001^***^	6.69 × 10^+11^
Confusion	Unrelated	0.51	0.09	0.93	0.15	3.346	.008^**^	8.12
	Insertion	−0.15	−0.28	−0.02	0.05	−3.107	.019^*^	2.94
Unrelated	Insertion	−0.66	−1.07	−0.25	0.15	−4.356	<.001^***^	125.11

The same conventions as in Table 1 are used here. * *P* ≤ .05, ** *P* ≤. 01, *** *P* ≤ .001

Taken together, the results of the manipulation check validated our manipulation of richness, demonstrating clear differences between the perceived richness of the intact and the blurred images, as expected. One might argue that these results are somewhat biased by task demands (i.e. participants equating richness with sharpness not because they perceive intact images as rich, but since they understand the logic behind the experiment). This concern is mitigated by two factors. First, the results of the first trial analysis demonstrate that this difference is found even before participants are familiarized with the stimuli. Second, the results of the image-based analysis show that intact versions of images were rated higher than their blurred counterparts. Furthermore, as seen in Fig. 10 (right), richness ratings were spread over a wide range even within the intact and the blurred conditions, with some overlap between them; if responses would solely reflect task demands, we would expect to see two clear clusters with all intact images rated high and all blurred images rated low. This is further reinforced by the fact that for some images, participants actually rated the blurred version as richer. Although this is only a minority group, it still demonstrates that participants did not simply equate “rich” with “intact.” Taken together, the data supports the assumption that the blur manipulation indeed degraded richness, in line with the previous literature (e.g. Prinz 2009, French 2015). This allowed us to test if the word IA measure would reflect these changes in perceived richness.

#### Direct replication

To test the correlation between word IA and mean confidence level, we performed a Bayesian Pearson correlation. Akin to the exploratory and preregistered studies, no correlation was found between words’ IA scores and their mean confidence rating [*r*(2702) = −0.019, *P* = .329, 95% CI = (−0.06, 0.02), BF_10_ = 0.039]. This lack of positive correlation is therefore consistent with the findings in the six main experiments, mitigating the concern that the lack of correlation stemmed from differences in instructions.

#### Long exposure

To test if the results in the main experiments changed when the images were presented for longer, we repeated the IA analysis. Even when the images were presented for 267 ms, word IAs of the intact images (*M* = 0.97, SD = 0.04) did not differ from those evoked by the black and white ones [*M* = 0.97, SD = 0.05; *t*(5781) = −0.485, *P* = 0.628, 95% CI = (−0.003, 0.002), BF_10_ = 0.03]. Thus, these experiments refuted the claim that the insensitivity of the IA measure to the richness manipulation hinged upon the short presentation durations.

## Discussion

Overall, our results suggest that the IA metric, derived from freely reported words, does not capture the richness of conscious perception (or lack thereof). This conclusion is supported by four types of evidence: first, the lack of correspondence between IA and perceptual richness, as found in the color manipulation, and to some degree in the blur manipulation; second, the consistent relationship between IA and vocabulary metrics; third, the failure to replicate the positive correlation between IA and confidence, originally claimed to demonstrate that IA captures an aspect of conscious perception; and fourth, the high rate of nonexisting words, out of which a non-negligible and consistent rate of insertion and confusion words was found.

The insensitivity of the IA metric to our manipulation of the richness of experience was found in two studies. Most consistent was the finding that colorless images elicited higher IA scores. This is perplexing if the measure indeed captures perception. Blurred images yielded mixed results: in the first study, IA scores did not differ between the blurred and the intact experiments. Notably, this result cannot be attributed to a lack of difference in the perceived richness of the intact and blurred experiences; in two control studies, we confirmed that the blur manipulation degraded the experienced stimulus. However, in the second, confirmatory, study, descriptions of blurred images had slightly lower IAs than intact ones. Thus, to the very least, one could claim that the IA measure yields inconsistent values with respect to the blurring manipulation. Conversely, one might disagree that blurring or grayscaling indeed reduces the richness of the image. We do not think that this is a very plausible concern: colorless images are clearly less rich than their colored counterparts, and blurred images were demonstrated to be less rich in two control experiments, in line with the literature (see Prinz 2009, French 2015, who deemed blurred and sharp experiences subjectively different).

The correlation between IA scores and frequency in English, found across all experiments, further questions the perceptual nature of the IA measure. This suggests that the IA scores might be at least partially driven by the uniqueness of the reported words. Similar effects of word frequency have typically been found in linguistic tasks (e.g. Oldfield and Wingfield 1965, Bonin et al. 2012). Thus, this finding suggests that the IA metric is more likely to reflect linguistic, rather than perceptual, information.

Our results also failed to replicate the original findings with respect to confidence ratings. In the original work, participants’ rated confidence positively correlated with the IA metric. The authors suggested that this serves as evidence for the measure being a proxy of conscious perception. This is because the correlation seemed to imply that participants had metacognitive access to their phenomenal experiences (Chuyin et al. 2022). However, in our case, across all six experiments, word IA did not positively correlate with confidence ratings. Notably, this failure to replicate was also found in a follow-up experiment that directly replicated the original work, with the very same instructions.

Finally, the more in-depth examination of the words revealed that across all experiments, many of the analyzed words did not describe items that appeared in the image (between 24% and 30% in the six experiments, meaning that 70%–76% of the words described items that did appear). Of the nonexisting words, many were conceptual, conveying observers’ affect or evaluation of the image (e.g. “cute”). This makes it harder to evaluate the extent to which these words reflect the actual perceived content, and they surely do not convey information about specific details in the image. Another important class of nonexisting words is the one describing items that did not appear in the image yet were compatible with its gist (insertion words, between 4% and 7%). This result suggests that participants might have relied on gist perception (Oliva 2005) when performing the task in one of two possible ways: first, at the cognitive level, the short presentation allowed them to grasp the meaning of the scene and then possibly guess which objects should appear in it, even if they had not seen them. Such reliance on unexpected strategies is a known phenomenon that has been reported in other tasks, leading to erroneous conclusions; for example, in the field of unconscious processing, a highly influential work by Kouider and Dupoux (2004) has shown that participants can be at chance at recognizing masked words while still having partial awareness of some of the letters. This awareness can then drive performance to evoke behavior that mimics semantic priming effects, even for meaningless words that share some letters with semantically congruent ones.

In the IA case, participants could have relied on some crude perception of the gist to generate behavior that is compatible with a much richer experience. This interpretation aligns with similar claims about reports of specific details that were actually driven by gist in the context of the richness of conscious perception (Irvine 2011). Presumably, some words that participants gave reflected the expected objects that should have appeared in the scene. When this was actually the case (which is likely as these expectations are based on these objects typically appearing in these contexts), there was a match between the responses and the contents of the scene. Yet, in some cases, there was no such match, leading to insertion words. Thus, the fact that such words were consistently given across all experiments provides substantial support to this alternative interpretation.

One could still argue, however, that these insertion words reflect false perceptions rather than a cognitive strategy. According to this interpretation, participants truly reported the content of their perception, yet that perception was not veridical (Ramachandran 1993, Seth 2021, Levinson and Baillet 2022). Indeed, gist processing affects object recognition (Bar 2004, Tal and Bar 2014, Truman and Mudrik 2018), especially when the object itself is ambiguous (Oliva and Torralba 2007, Brandman and Peelen 2017). Further support to this claim can be found in a recent study where participants failed to notice incongruent objects when scenes were presented for short durations, arguably “replacing” them with scene-congruent objects (Biderman and Mudrik 2018; see also Qianchen et al. 2022). Therefore, under the perceptual interpretation, the IA measure indeed captures the content of perception, which is not always veridical. Our data does not allow us to arbitrate between these two interpretations. However, the finding of higher confidence ratings for existing words compared to nonexisting ones seems to go against this interpretation. If observers were simply reporting what they perceptually experienced in all cases, there should not have been any effect of confidence when analyzing the words based on their compatibility with the scene—for the participants, it should not have made a difference. Yet, we found that words describing items that did exist in the scenes evoked higher confidence ratings. This finding aligns with the hypothesis that at least some of these nonexisting words were not based on what participants actually perceived, but rather generated using other strategies. Thus, both the existence of the insertion words and the difference in confidence between existing words and all types of nonexisting words cast doubt on the claim that the IA measure indeed captures the content of perception. Further research is accordingly needed to exclude the task demands, strategy-based, account.

An additional, more conceptual, claim can also be raised with respect to the IA measure. As the measure focuses on the uniqueness of the provided words, given a highly diverse stimulus set, it heavily depends on the similarity of the images in the set. For example, suppose we used an alternative stimulus set, where all images are pictures of the Eiffel Tower, but from very different angles, distances, and so on, such that these images lead to qualitatively different conscious experiences. In this case, we would expect relatively low word IAs because the provided words would likely be similar across images (e.g. Eiffel Tower, Paris, and park) despite the qualitatively different phenomenal experiences they evoke. Crucially, however, the low word IAs of these words would not necessarily imply that the reported items were not experienced in full, including fine details. Each individual experience could have been immensely rich, but the measure would probably imply otherwise, given the similarity of the stimuli. The main point of this example is that the specificity and detail of the contents of one’s experience of a specific image is not more or less rich depending on the rest of the stimulus set—while this is not true for the word IA metric. This again demonstrates how the word IA measure can be dissociated from the content of conscious perception.

Taken together, these findings raise a more general concern about the suitability of language-based measures for probing the richness of conscious perception. Many supporters of the richness hypothesis tend to contrast experience with cognition, claiming that perception overflows other cognitive processes (Block 2011) or that the two should be clearly delineated (Block 2023). Yet, verbal report–based measures use language, and in a way thinking, to capture the content of experience. In some sense, this seems to be contradictory: if perception overflows language, how can we use language to measure the richness of conscious perception? We suggest here that doing so inevitably introduces the characteristics (and limitations) of language (e.g. its sequential nature, which poses memory limitations: Kouider et al. 2010), getting us farther away from the object of interest.

This does not mean that the attempt to quantify the richness of perception is necessarily moot. Alternative reporting techniques might provide insight into observers’ experiences: For example, using drawing as a measure (for a review, see Fan et al. 2023) can be instrumental for tracking experience, as previously suggested by Haun et al. (2017). This approach relies primarily on metrics derived from scoring line drawings to compare the content of perception and memory under different viewing conditions (e.g. free viewing vs. recall: Bainbridge et al. 2019; foveal vs. peripheral viewing: Coates et al. 2017). However, these studies might suffer from similar limitations, as even in drawings, participants might rely on abstraction and conceptual knowledge (Fan et al. 2023), potentially moving away from the actual content of conscious experience. Alternatively, multimodal methods combining reports with physiological and neural data might be better equipped to provide more direct information about the perceived content, similar to no-report paradigms (Tsuchiya et al. 2015). Thus, more efforts are needed to get us closer to developing a good enough measure of conscious perception and its richness.

Whichever measure is suggested as a new means to study the content and richness of perception, it would have to be convincingly validated, like is typically done when psychological measures are developed (e.g. for emotion: Beck et al. 1988 or working memory: Pomplun and Custer 2005). However, validation seems to be more challenging in the current case because we do not have access to the “ground truth,” that is, to the actual content of one’s experience (Nagel 1980, 1974; but see Dennett 1988). In a way, this is one of the greatest challenges the field of consciousness has been struggling with since its inception (Reingold and Merikle 1988, Sandberg et al. 2010). Here, we suggest a possible way to meet this challenge or at least mitigate it. Clearly, we cannot directly test the suggested measure against the actual content of perception in its entirety. Instead, we have tried here to take a simpler approach and crudely manipulate certain aspects of the content of perception, which should be captured by the suggested measure. Therefore, if a measure is held to index the content (and, potentially, richness) of experience, it should reflect changes in content and richness. This logic has historically guided scientists when designing metrics for constructs such as the hardness of metals (Tabor 1951) or conservation of mass (in the experiments of Antoine Lavoisier and as described in Holmes 1987). Going back to the work on partial awareness (Kouider and Dupoux 2004), there too, the authors manipulated the semantic content of the masked words and showed that the measure of the so-called “semantic” priming is not sensitive to that change. Such disconfirmatory experiments (Firestone and Scholl 2016) are very instrumental in testing and validating possible measures. We accordingly suggest adopting this approach when developing measures of experienced content, as we have done here.

## Conclusion

In this work, we tested a recently suggested measure of conscious perception—word IA (Chuyin et al. 2022). This measure was originally suggested to solve a timely and important challenge, given the limitations of previous paradigms to fully capture the contents of perception (Haun et al. 2017). It incorporates an innovative approach by trying to quantify, for the first time, the level of agreement between participants around the content of conscious perception, in a way that emphasizes the specificity of that content. However, our experiments revealed that this agreement might not index perception itself, but rather stem from cognitive-based strategies or linguistic regularities. The results further demonstrated that the measure is not sensitive to large differences in perceived richness, questioning the construct validity of this measure. This calls for developing new measures, which could be tested and validated using a similar rationale as the one taken here.

## Data Availability

All experiments’ running codes, collected data, and analysis codes are available on Open Science Framework (OSF): osf.io/r73kp/. The registration of the preregistered study is on OSF as well: osf.io/2y3gr.

## References

[R1] Adobe Inc . *Adobe Photoshop* [Computer software]. 2019. https://www.adobe.com/products/photoshop.html (28 August 2024, date last accessed).

[R2] Bainbridge WA, Hall EH, Baker CI. Drawings of real-world scenes during free recall reveal detailed object and spatial information in memory. *Nat Commun* 2019;10:5. doi: 10.1038/s41467-018-07830-6 PMC6315028 30602785

[R3] Bar M . Visual objects in context. *Nat Rev Neurosci* 2004;5:617–29. doi: 10.1038/nrn1476 15263892

[R4] Beck AT, Steer RA, Carbin MG. Psychometric properties of the Beck Depression Inventory: twenty-five years of evaluation. *Clin Psychol Rev* 1988;8:77–100. doi: 10.1016/0272-7358(88)90050-5

[R5] Biderman N, Mudrik L. Evidence for implicit—but not unconscious—processing of object-scene relations. *Psychol Sci* 2018;29:266–77. doi: 10.1177/0956797617735745 29283750

[R6] Block N . Consciousness, accessibility, and the mesh between psychology and neuroscience. *Behav Brain Sci* 2007;30:481–548. doi: 10.1017/S0140525X07002786 18366828

[R7] Block N . Consciousness and cognitive access. *Proc Aristotelian Soc* 2008;108:289–317. doi: 10.1111/j.1467-9264.2008.00247.x

[R8] Block N . Perceptual consciousness overflows cognitive access. *Trends Cogn Sci* 2011;15:567–75. doi: 10.1016/j.tics.2011.11.001 22078929

[R9] Block N . Rich conscious perception outside focal attention. *Trends Cogn Sci* 2014;18:445–7. doi: 10.1016/j.tics.2014.05.007 24890010

[R10] Block N . *The Border between Seeing and Thinking*, 1st edn. New York, NY: Oxford University Press, 2023.

[R11] Bonin P, Roux S, Barry C et al. Evidence for a limited-cascading account of written word naming. *J Exp Psychol* 2012;38:1741–58. doi: 10.1037/a0028471 22612168

[R12] Brandman T, Peelen MV. Interaction between scene and object processing revealed by human fMRI and MEG decoding. *J Neurosci* 2017;37:7700–10. doi: 10.1523/JNEUROSCI.0582-17.2017 28687603 PMC6596648

[R13] Bridges D, Pitiot A, MacAskill MR et al. The timing mega-study: comparing a range of experiment generators, both lab-based and online. *PeerJ* 2020;8:e9414. doi: 10.7717/peerj.9414 PMC7512138 33005482

[R14] Bronfman ZZ, Brezis N, Jacobson H et al. We see more than we can report. *Psychol Sci* 2014;25:1394–403. doi: 10.1177/0956797614532656 24815608

[R15] Bronfman ZZ, Jacobson H, Usher M. Impoverished or rich consciousness outside attentional focus: recent data tip the balance for overflow. *Mind Language* 2019;34:423–44. doi: 10.1111/mila.12217

[R16] Brysbaert M, New B. Moving beyond Kučera and Francis: a critical evaluation of current word frequency norms and the introduction of a new and improved word frequency measure for American English. *Behav Res Methods* 2009;41:977–90. doi: 10.3758/BRM.41.4.977 19897807

[R17] Chuyin Z, Koh ZH, Gallagher R et al. What can we experience and report on a rapidly presented image? Intersubjective measures of specificity of freely reported contents of consciousness. *F1000Research* 2022;11:69. doi: 10.12688/f1000research.75364.1 PMC9493396 36176545

[R18] Coates DR, Wagemans J, Sayim B. Diagnosing the periphery: using the Rey–Osterrieth complex figure drawing test to characterize peripheral visual function. *i-Perception* 2017;8:2041669517705447. doi: 10.1177/2041669517705447 PMC5453411 28607664

[R19] Cohen MA, Botch TL, Robertson CE. The limits of color awareness during active, real-world vision. *Proc Natl Acad Sci USA* 2020;117:13821–7. doi: 10.1073/pnas.1922294117 32513698 PMC7306755

[R20] Cohen MA, Dennett DC. Consciousness cannot be separated from function. *Trends Cogn Sci* 2011;15:358–64. doi: 10.1016/j.tics.2011.06.008 21807333

[R21] Cohen MA, Dennett DC, Kanwisher N. What is the bandwidth of perceptual experience? *Trends Cogn Sci* 2016;20:324–35. doi: 10.1016/j.tics.2016.03.006 27105668 PMC4898652

[R22] Cornoldi C De Beni R Giusberti F et al. Chapter 20 The study of vividness of images. In: Logie RH, Denis M (eds.), *Advances in Psychology*, Vol. 80. North-Holland, Netherlands: Elsevier, 1991, 305–12.

[R23] Cronbach LJ, Meehl PE. Construct validity in psychological tests. *Psychol Bull* 1955;52:281–302. doi: 10.1037/h0040957 13245896

[R24] Dehaene S, Changeux JP, Naccache L et al. Conscious, preconscious, and subliminal processing: a testable taxonomy. *Trends Cogn Sci* 2006;10:204–11. doi: 10.1016/j.tics.2006.03.007 16603406

[R25] Dennett DC . Quining qualia. In: Marcel AJ, Bisiach E (eds.), *Consciousness in Modern Science*. New York: Oxford University Press, 1988, 42–77.

[R26] Fan JE, Bainbridge WA, Chamberlain R et al. Drawing as a versatile cognitive tool. *Nat Rev Psychol* 2023;2:556–68. doi: 10.1038/s44159-023-00212-w 39239312 PMC11377027

[R27] Firestone C, Scholl BJ. Cognition does not affect perception: evaluating the evidence for “top-down” effects. *Behav Brain Sci* 2016;39:e229. doi: 10.1017/S0140525X15000965 26189677

[R28] French C . Naive realist perspectives on seeing blurrily. In: Stazicker J (ed.), *The Structure of Perceptual Experience*. 1st edn. Malden, MA: John Wiley & Sons, 2015: 31–51.

[R29] Haun AM . What is visible across the visual field? *Neurosci Conscious* 2021;2021:niab006. doi: 10.1093/nc/niab006 PMC8167368 34084558

[R30] Haun AM, Tononi G, Koch C et al. Are we underestimating the richness of visual experience? *Neurosci Conscious* 2017;2017:niw023. doi: 10.1093/nc/niw023 PMC6007133 30042833

[R31] Hawkins B, Evans D, Preston A et al. Color diversity judgments in peripheral vision: evidence against “cost-free” representations. *PLoS One* 2022;17:e0279686. doi: 10.1371/journal.pone.0279686 PMC9803108 36584092

[R32] Holmes FL . *Lavoisier and the Chemistry of Life: An Exploration of Scientific Creativity (Repr.)*. Madison, WI, USA: University of Wisconsin Press, 1987.

[R33] Honnibal M, Montani I. spaCy 2: natural language understanding with bloom embeddings, convolutional neural networks and incremental parsing. In Press 2017;:411–20.

[R34] Irvine E . Rich experience and sensory memory. *Philos Psychol* 2011;24:159–76. doi: 10.1080/09515089.2010.543415

[R35] Jackson-Nielsen M, Cohen MA, Pitts MA. Perception of ensemble statistics requires attention. *Conscious Cogn* 2017;48:149–60. doi: 10.1016/j.concog.2016.11.007 27918894

[R36] JASP Team . JASP (Version 0.16.3) [Computer software]. 2022. https://jasp-stats.org/ (28 August 2024, date last accessed).

[R37] Kouider S, De Gardelle V, Sackur J et al. How rich is consciousness? The partial awareness hypothesis. *Trends Cogn Sci* 2010;14:301–7. doi: 10.1016/j.tics.2010.04.006 20605514

[R38] Kouider S, Dupoux E. Partial awareness creates the “Illusion” of subliminal semantic priming. *Psychol Sci* 2004;15:75–81. doi: 10.1111/j.0963-7214.2004.01502001.x 14738512

[R39] Landman R, Spekreijse H, Lamme VAF. Large capacity storage of integrated objects before change blindness. *Vis Res* 2003;43:149–64. doi: 10.1016/S0042-6989(02)00402-9 12536137

[R40] Levinson M, Baillet S. Perceptual filling-in dispels the veridicality problem of conscious perception research. *Conscious Cogn* 2022;100:103316. doi: 10.1016/j.concog.2022.103316 35358869

[R41] Li FF, VanRullen R, Koch C et al. Rapid natural scene categorization in the near absence of attention. *Proc Natl Acad Sci USA* 2002;99:9596–601. doi: 10.1073/pnas.092277599 12077298 PMC123186

[R42] Maier A, Tsuchiya N. Growing evidence for separate neural mechanisms for attention and consciousness. *Atten Percept Psychophys* 2021;83:558–76. doi: 10.3758/s13414-020-02146-4 33034851 PMC7886945

[R43] McClelland T, Bayne T. Ensemble coding and two conceptions of perceptual sparsity. *Trends Cogn Sci* 2016;20:641–2. doi: 10.1016/j.tics.2016.06.008 27422442

[R44] Michel M . Consciousness science underdetermined: a short history of endless debates. *Ergo Open Access J Philos* 2019;6:771–809. doi: 10.3998/ergo.12405314.0006.028

[R45] Nagel T . What is it like to be a bat? *Philos Rev* 1974;83:435–50. doi: 10.2307/2183914

[R46] Nagel T . 14. Armstrong on the mind. In: Block N (ed.), *The Language and Thought Series*. Vol. 1. Cambridge, MA and London, England: Harvard University Press, 1980, 200–6.

[R47] Noah S, Mangun GR. Recent evidence that attention is necessary, but not sufficient, for conscious perception. *Ann NY Acad Sci* 2020;1464:52–63. doi: 10.1111/nyas.14030 30883785

[R48] Oldfield RC, Wingfield A. Response latencies in naming objects. *Q J Exp Psychol* 1965;17:273–81. doi: 10.1080/17470216508416445 5852918

[R49] Oliva A . Gist of the scene. In: Itti L, Rees G, Tsotsos JK (eds.), *Neurobiology of Attention*. Cambridge, MA: Academic Press, 2005, 251–6.

[R50] Oliva A, Torralba A. The role of context in object recognition. *Trends Cogn Sci* 2007;11:520–7. doi: 10.1016/j.tics.2007.09.009 18024143

[R51] Peirce J, Gray JR, Simpson S et al. PsychoPy2: experiments in behavior made easy. *Behav Res Methods* 2019;51:195–203. doi: 10.3758/s13428-018-01193-y 30734206 PMC6420413

[R52] Phillips I . Perception and iconic memory: what sperling doesn’t show. *Mind Language* 2011;26:381–411. doi: 10.1111/j.1468-0017.2011.01422.x

[R53] Phillips I . No watershed for overflow: recent work on the richness of consciousness. *Philos Psychol* 2016;29:236–49. doi: 10.1080/09515089.2015.1079604

[R54] Pomplun M, Custer M. The construct validity of the stanford-binet 5 measures of working memory. *Assessment* 2005;12:338–46. doi: 10.1177/1073191105276796 16123254

[R55] Prinz J . Is consciousness embodied. In: Robbins P, Aydede M (eds.), *The Cambridge Handbook of Situated Cognition*. Cambridge, UK: Cambridge University Press, 2009, 419–36.

[R56] Qianchen L, Gallagher RM, Tsuchiya N. How much can we differentiate at a brief glance: revealing the truer limit in conscious contents through the massive report paradigm (MRP). *Royal Soc Open Sci* 2022;9:210394. doi: 10.1098/rsos.210394 PMC9128849 35619998

[R57] Ramachandran VS . Interactions between motion, depth, color and form: the utilitarian theory of perception. In: Blakemore C, Adler K, Pointon M (eds.), *Vision: Coding and Efficiency*. Cambridge, UK: Cambridge University Press, 1993, 346–60.

[R58] Reingold EM, Merikle PM. Using direct and indirect measures to study perception without awareness. *Percept Psychophys* 1988;44:563–75. doi: 10.3758/BF03207490 3200674

[R59] Rensink RA, O’Regan JK, Clark JJ. To see or not to see: the need for attention to perceive changes in scenes. *Psychol Sci* 1997;8:368–73. doi: 10.1111/j.1467-9280.1997.tb00427.x

[R60] Sandberg K, Timmermans B, Overgaard M et al. Measuring consciousness: is one measure better than the other? *Conscious Cogn* 2010;19:1069–78. doi: 10.1016/j.concog.2009.12.013 20133167

[R61] Seth AK . *Being You: A New Science of Consciousness*. New York: Dutton, 2021.

[R62] Simons DJ, Chabris CF. Gorillas in our midst: sustained inattentional blindness for dynamic events. *Perception* 1999;28:1059–74. doi: 10.1068/p281059 10694957

[R63] Simons DJ, Franconeri SL, Reimer RL. Change blindness in the absence of a visual disruption. *Perception* 2000;29:1143–54. doi: 10.1068/p3104 11220207

[R64] Sligte IG, Scholte HS, Lamme VAF. Are there multiple visual short-term memory stores? *PLoS One* 2008;3:e1699. doi: 10.1371/journal.pone.0001699 PMC2246033 18301775

[R65] Sperling G . The information available in brief visual presentations. *Psychol Monogr* 1960;74:1. doi: 10.1037/h0093759

[R66] Stazicker J . Attention, visual consciousness and indeterminacy. *Mind Language* 2011;26:156–84. doi: 10.1111/j.1468-0017.2011.01414.x

[R67] Tabor D . *The Hardness of Metals*. Oxford, England: Clarendon Press, 1951.

[R68] Tal A, Bar M. The proactive brain and the fate of dead hypotheses. *Front Comput Neurosci* 2014;8:138. doi: 10.3389/fncom.2014.00138 PMC4219452 25408645

[R69] Truman A, Mudrik L. Are incongruent objects harder to identify? The functional significance of the N300 component. *Neuropsychologia* 2018;117:222–32. doi: 10.1016/j.neuropsychologia.2018.06.004 29885960

[R70] Tsuchiya N, Wilke M, Frässle S et al. No-report paradigms: extracting the true neural correlates of consciousness. *Trends Cogn Sci* 2015;19:757–70. doi: 10.1016/j.tics.2015.10.002 26585549

[R71] Vandenbroucke ARE, Sligte IG, Fahrenfort JJ et al. Non-attended representations are perceptual rather than unconscious in nature. *PLoS One* 2012;7:e50042. doi: 10.1371/journal.pone.0050042 PMC3507828 23209639

[R72] Ward EJ, Bear A, Scholl BJ. Can you perceive ensembles without perceiving individuals?: the role of statistical perception in determining whether awareness overflows access. *Cognition* 2016;152:78–86. doi: 10.1016/j.cognition.2016.01.010 27038156

[R73] Wolfe JM . Inattentional amnesia. *Fleeting Memories* 1999;17:1–21.

[R74] Zeleznikow-Johnston A, Aizawa Y, Yamada M et al. Are color experiences the same across the visual field? *J Cogn Neurosci* 2023;35:509–42. doi: 10.1162/jocn_a_01962 36638234

